# Distribution of HLA-A, -B, and -C Alleles and HLA/KIR Combinations in Han Population in China

**DOI:** 10.1155/2014/565296

**Published:** 2014-06-09

**Authors:** Yunsong Shen, Danfeng Cao, Yalin Li, J. K. Kulski, Lei Shi, Hongjun Jiang, Qianli Ma, Jiankun Yu, Jingxian Zhou, Yufeng Yao, Li Shi

**Affiliations:** ^1^The First People's Hospital in Yunnan Province and The Affiliated Hospital of Kunming Science and Technology University, Kunming 650032, China; ^2^Yunnan Key Laboratory of Vaccine Research and Development on Severe Infectious Diseases, Institute of Medical Biology, Chinese Academy of Medical Sciences (CAMS) and Peking Union Medical College (PUMC), Kunming 650118, China; ^3^The Centre for Forensic Science, University of Western Australia, Nedlands, WA 6009, Australia; ^4^The No. 3 Affiliated Hospital of Kunming Medical University, Kunming 650118, China

## Abstract

We investigated polymorphisms of the human leukocyte antigen (HLA) class I (A, B, and C) loci of a Han population (*n*, 239) from the Yunnan province, Southwest China, using high-resolution polymerase chain reaction-Luminex (PCR-Luminex) typing. We combined the HLA data from this study with the KIR genotypes from a previous study of this Han population to analyze the combination of KIR/HLA ligands. A total of 27 HLA-A, 54 HLA-B, and 31 HLA-C alleles were found in this population. The frequencies of A*11:01, A*24:02, B*40:01, B*46:01, C*01:02, C*03:04, and C*07:02 were all > 10%. The following haplotypes were common, with frequencies > 5%: 1 A-B (A*02:07-B*46:01), 2 A-C (A*02:07-C*01:02, and A*11:01-C*07:02), 4 C-B (B*13:01-C*03:04, B*40:01-C*07:02, B*46:01-C*01:02 and B*58:01-C*03:02), and 1 A-C-B (A*02:07-C*01:02-B*46:01). Analysis of KIR3D and their ligands HLA-A3/A11 and HLA-Bw4 showed that the frequencies of 3DL2^+^-A3/A11^+^ and 3DL2^+^-A3/A11^−^ were 0.527 and 0.473, and the frequencies of 3DL1^+^-Bw4^+^, 3DL1^+^-Bw4^−^, 3DL1^−^-Bw4^+^, and 3DL1^−^-Bw4^−^ were 0.552, 0.397, 0.038, and 0.013, respectively. The results of KIR/HLA-C combination analysis showed that all individuals had at least one inhibitory or activating KIR/HLA-C pair, and one KIR/HLA-C pair was the most frequent (157/239), followed by two pairs (46/239), three pairs (33/239), and no pairs (3/239). Comparison of KIR gene and HLA gene and their pair frequency between Yunnan Han and the isolated Han (FYDH) who also lived in Yunnan province showed no significant difference (*P* > 0.05) in KIR frequencies, but significant differences (*P* < 0.05) for some HLA allele frequencies. In addition, there was no significant difference (*P* > 0.05) between the two populations for KIR/HLA pairs.

## 1. Introduction 

The major histocompatibility complex (MHC) genomic region or human leukocyte antigen (HLA) super-locus at 6p21 encodes at least 132 protein-coding genes, including six classical transplantation HLA genes with multiple roles in immune regulation [1]. The high degree of diversity of alleles and haplotypes makes the classical HLA loci particularly valuable for investigating the origins and migration of human populations and for disease association studies.

Killer cell immunoglobulin-like receptors (KIRs), which are found on natural killer (NK) cells, have a central role in immune responses [2]. The KIR genes are located on chromosome 19q13.4 within the leukocyte receptor complex, which is clustered in one of the most variable regions of the human genome and shows extensive polymorphism in terms of both gene content and sequence polymorphism [2, 3].

HLA class I molecules and the KIR serve as ligand and receptor. Certain combinations of KIRs and HLA molecules can transmit activating or inhibitory signals to regulate the function of NK cells [4]. For HLA-A molecules, several studies have reported that KIR3DL2 interacted with HLA-A3 and -A11 allotypes [5–7]. For HLA-B molecules, the KIR3DL1 interacts with HLA-B Bw4 allotypes [6, 8, 9], and no KIRs have been found to bind the HLA-B Bw6 allotypes with high affinity [6, 10]. For HLA-C molecules, on the basis of a dimorphism (asparagine or lysine) at position 80 in the alpha helix, HLA-C alleles can be placed into one of two categories, C1 and C2, which are recognized differentially by the KIR receptors. NK cells that express KIR2DL2 and KIR2DL3 interact with HLA-C1 allotypes, whereas KIR2DL1 and KIR2DS1 interact with HLA-C2 allotypes [11, 12].

Chinese populations consist of 56 ethnic groups and Han is the largest population (~93%). The Han were derived from the Huaxia tribe living in the area of the Yellow River, who then migrated to the area of the Yangtze River and have now spread throughout the country.

On the basis of HLA-A, -B, and -DRB1 allele frequencies, our earlier work showed that the Han population can be divided into Southern and Northern Han [13]. We previously reported the distribution of HLA-A, -B, and -DRB1 genes and haplotypes at low resolution in a Han population (*n*, 129) from the Yunnan province (YNH) and found that it had characteristics of both northern and southern Chinese groups based on HLA data [13]. In current study, we investigated polymorphisms of HLA class I (A, B, and C) loci of a Han population from Yunnan (*n*, 239) using high-resolution polymerase chain reaction-Luminex (PCR-Luminex) typing to get the high-resolution HLA data of YNH. In addition, we combined the HLA data with KIR genotypes of the YNH to analyze the combination of KIR/HLA pairs and looked for any difference between the YNH and an isolated Han subpopulation [14–16] that lived in the Fenyandong (FYDH) region in Yunnan province.

## 2. Materials and Methods

### 2.1. Participants

A total of 239 unrelated individuals, which were from the Yunnan Province of Southwest of China (YNH), were included in this study and the geographic origin, nationality, and pedigree (unrelated through at least three generations) of each individual were ascertained before sampling. All individuals were healthy and provided informed consent.

### 2.2. HLA Typing

Blood samples were collected and genomic DNA was extracted from peripheral lymphocytes using the QIAamp Blood Kit (Qiagen, Hilden, Germany) according to the manufacturer's protocol. DNA-based HLA typing was done with the Luminex multianalyte profiling system (xMAP) using the WAKFlow HLA typing kit (Wakunaga, Hiroshima, Japan).

### 2.3. KIR Typing

The results of the KIR typing in the Yunnan Han population are published [16] and here we have used the KIR results to analyze their relationship with HLA-A3/A11, HLA-Bw4, and the HLA-C1 and HLA-C2 groups that we obtained in this study.

### 2.4. Statistical Analysis

Allele and inferred haplotype frequencies (deduced from the phenotype) were calculated by the expectation-maximization algorithm in the Pypop software on the basis of the genotyping results [17, 18]. The HLA loci observed and expected heterozygosity were calculated by using MolKin v3.0 software [19]. For each locus, the Hardy-Weinberg equilibrium was assessed using the Guo and Thompson method [20] and the Ewens-Watterson homozygosity test of neutrality was done [21, 22]. Linkage disequilibrium (LD) was analyzed using the Pypop software [17, 18]. The HLA-A3/A11 and HLA-Bw4 groups were used to analyze KIR/HLA combinations. The HLA-C1 and HLA-C2 groups were also used to analyze KIR/HLA combinations. The presence of two inhibitory (2DL2/2DL3+HLA-C1 and 2DL1+HLA-C2) and one activating (2DS1+HLA-C2) receptor-ligand pairs was evaluated for each individual. The differences in significance between the HLA/KIR pair frequencies were determined by a contingency test. Statistical significance was defined at the 5% level.

## 3. Results 

### 3.1. Hardy-Weinberg's Equilibrium Observed Heterozygosity and Expected Heterozygosity and Ewens-Watterson Test of Neutrality for HLA-A, -B, and -C Loci

Table 1 showed the HLA-A, -B, and -C loci in Hardy-Weinberg's equilibrium (*P* > 0.05) and the results for the Evens-Watterson test of neutrality for HLA-A, -B, and -C loci in YNH. Data on HLA-A, -B, and -C did not reject the neutral model (*P* > 0.05). The observed and expected heterozygosity of HLA-A, -B, and -C loci were shown in Table 1.

### 3.2. HLA-A, -B, and -C Allele Frequencies

In all, 27 HLA-A, 54 HLA-B, and 31 HLA-C high resolution alleles were observed (Table 2). The three most common alleles at (1) HLA-A locus were 0.268 for HLA-A*11:01, 0.190 for HLA-A*24:02, and 0.094 for HLA-A*02:07; (2) HLA-B locus were 0.144 for HLA-B*46:01, 0.117 for HLA-B*40:01, and 0.084 for HLA-B*13:01; and (3) HLA-C locus were 0.203 for HLA-C*01:02, 0.153 for HLA-C*07:02, and 0.117 for HLA-C*03:04.

Compared with HLA alleles of an isolated Han population (FYDH) in Yunnan province [14], the frequencies of some alleles were significantly different (*P* < 0.05) between these two Han populations. On the HLA-A locus, the frequencies of A*02:01 and A*02:07 were 0.086 and 0.094, respectively, in YNH and 0.010 and 0.183, respectively, in FYDH. On the HLA-B locus, B*40:01 was present in YNH at frequencies of >10% (0.117) but were observed only at frequencies of 0.045 in FYDH. By contrast, the common allele of HLA-B*15:02 in FYDH (0.124) was present at a low frequency in YNH (0.044). At the HLA-C locus, HLA-C*07:02 was more common in YNH compared to FYDH (0.153 versus 0.094), whereas HLA-C*08:01 was more common in FYDH compared to YNH (0.149 versus 0.040).

### 3.3. The Linkage Disequilibrium of HLA-A, -B, and -C Loci and the Haplotype Frequencies

The likelihood ratio test of linkage disequilibrium found that all pairwise associations (Table 3) were statistically significant (*P* < 0.001). Therefore, we estimated the HLA haplotype frequencies bearing 2-3 loci and show the 2-3 loci haplotype frequencies of >1% in Table 4. The most common A-B haplotype was A*02:07-B*46:01 (0.073), the most common A-C haplotypes were A*02:07-C*01:02 (0.068) and A*11:01-C*07:02 (0.070), and the most common C-B haplotypes were B*13:01-C*03:04 (0.071), B*40:01-C*07:02 (0.052), B*46:01-C*01:02 (0.132), and B*58:01-C*03:02 (0.054). The most common A-C-B haplotype was A*02:07-C*01:02-B*46:01 (0.067).

The difference between FYDH and YNH for the HLA was obvious also at the haplotype level [14]. A*11:01-C*08:01-B*15:02 was a common haplotype in FYDH (0.124), but the frequency of this haplotype was only 0.014 in YNH. Similarly, the frequency of A*02:07-C*01:02-B*46:01 was 0.126 in FYDH but 0.067 in YNH, and the frequency of A*33:03-C*03:02-B*58:01 was 0.074 in FYDH but 0.042 in YNH.

### 3.4. Two KIR3D and Their Ligands HLA-A3 and -A11

The distribution of two KIR3D genes and their HLA-A3/A11 ligands is given in Table 5. In all, the frequencies of 3DL2^+^-A3/A11^+^ and 3DL2^+^-A3/A11^+^ were 0.527 and 0.473 in YNH. No significant differences (*P* > 0.05) were detected in the statistical comparison between the YNH and the FYDH.

### 3.5. Two KIR3D and Their Ligands HLA-Bw4

The distribution of two KIR3D genes and their HLA-Bw4 ligands is given in Table 6. In all, the frequencies of 3DL1^+^-Bw4^+^, 3DL1^+^-Bw4^−^, 3DL1^−^-Bw4^+^, and 3DL1^−^-Bw4^−^ were 0.552, 0.397, 0.038, and 0.013, respectively in YNH. There was no statistical difference (*P* > 0.05) in the frequencies between the YNH and FYDH populations.

### 3.6. Combinations of KIR/HLA-C

The HLA and KIR genes segregate on two different chromosomes but have a functional correlation owing to the KIR/HLA-C ligand-receptor pairs. Using our KIR data from earlier studies [15], we analyzed two inhibitory (2DL2/2DL3+HLA-C1 and 2DL1+HLA-C2) and one activating (2DS1+HLA-C2) receptor-ligand pairs (Table 7). First, we analyzed inhibitory and activating KIR/HLA-C pairs in YNH. We observed that all individuals had at least one inhibitory or activating KIR/HLA-C pair. One KIR/HLA-C pair was the most frequent (157/239), followed by two pairs (46/239), three pairs (33/239), and no pairs (3/239). Second, we differentiated between inhibitory and activating KIR/HLA-C pairs. The frequencies for different numbers of inhibitory KIR/HLA-C pairs in YHN were 75/239 for two inhibitory KIR/HLA-C pairs (KIR2DL2/2DL3+HLA-C1, KIR2DL1+HLA-C2), 161/239 for one inhibitory KIR/HLA-C pair (152 KIR2DL2/2DL3+HLA-C1 and 9 KIR2DL1+HLA-C2), and three for no pairs (3/239). For the activating KIR/HLA-C pairs, 37/239 were for one KIR/HLA-C pair (35 KIR2DS1+HLA-C2) and 202/239 were for no pairs. There was no statistical difference (*P* > 0.05) in the frequencies of KIR/HLA-C ligand-receptor pairs between the YNH and FYDH populations.

## 4. Discussion

Earlier, we reported the distribution of HLA-A, -B, and -DRB1 genes and haplotypes at low resolution in a Yunnan Han (YNH) population and found that it had characteristics of both northern and southern Chinese groups, although they live in the southwest of China [13]. In the present study, we genotyped the HLA-A, HLA-B, and HLA-C genes at high resolution in YNH and compared their HLA alleles, haplotypes, and combinations of KIR/HLA to an isolated Han subpopulation [14–16] that lived in the Fenyandong (FYDH) region of the Yunnan province.

The FYDH subpopulation became geographically isolated and lived in the FYDH regionof Yunnan province (Southwest China) after the group had emigrated from other places more than 200 years ago [23]. Since then, the FYDH did not usually communicate with other groups, as it took at least one day to walk to the nearest town. Our previous studies showed that the FYDH has a particular distribution of HLA alleles and KIR genotypes, and there is a difference between the YNH and FYDH populations in the distribution of HLA-A and -B alleles at low resolution [14]. In the present study, a significant difference was observed between the YNH and FYDH for the high resolution, HLA allele frequencies. Firstly, the number of alleles was less in the FYDH than the YNH population. Secondly, the HLA alleles of A*11:01 (0.317), A*02:07 (0.183), B*15:02 (0.124), B*46:01 (0.198), C*08:01 (0.149), and C*07:02 (0.094) were at a substantially higher frequency in the FYDH subpopulation than in the YNH (Table 2) or in other Chinese Han populations. Thirdly, the frequencies of common haplotypes such as A*02:07- C*01:02-B*46:01 (0.126 vs 0.067) and A*11:01-C*08:01-B*15:02 (0.124 vs 0.014) were higher in the FYDH than in the YNH, respectively. Therefore, the high frequencies of some of these alleles in the FYDH could be a reflection of genetic drift, founder effects, and/or population bottlenecks [24, 25], suggesting that genetic drift and/or selection and subsequent geographic isolation has played a more important role in distribution of HLA alleles in FYDH [14] than the YNH.

By contrast, comparison of KIR frequencies between YNH and FYDH showed that the two populations were more similar for the KIR genes than the HLA genes [15, 16]. Firstly, the 15 KIR genes frequencies in YNH did not differ significantly compared to FYDH except for KIR2DS5 (*P* < 0.05). Secondly, more than 40 KIR genotypes showed no significant difference between YNH and FYDH, except for genotype 2 and genotype 33, respectively, and the KIR AA genotype was the most frequent in YNH and FYDH (0.478 and 0.570, resp.). Thirdly, the similar ratio of A and B haplotype frequencies in YNH and FYDH was observed (2.39 : 1 vs 2.91 : 1, resp.) [15, 16]. The KIR genes, genotypes, and haplotypes in Han populations were the same as those in Asian populations but were different from those in African and Caucasian populations [26–30]. Moreover, the significant difference of two activating genes (KIR2DS4 and 2DS3) and one inhibitory gene (KIR2DL2) that was observed between northern and southern Han did not show any significant difference between the YNH and FYDH in the present study.

When the HLA/KIR combination was compared, there was still no significant difference between these two Han populations. Individuals with KIR3DL2 genes coupled with HLA-A3/A11 were predominant in YNH and FYDH with similar frequencies. Individuals with KIR3DL1 genes coupled with HLA-Bw4 were predominant in YNH and FYDH with similar frequencies. Individuals that did not have the KIR3DL1 genes coupled with HLA-Bw4 were also common in the YNH and the FYDH. When the HLA-C1/C2 ligands were considered, the frequencies of the individuals with HLA-C1 homozygosity coupled to the KIR2DLl and KIR2DL2/2DL3 genes (but lacking the KIR2DSl gene) were predominant in the YNH and the FYDH with similar frequencies around 50% (data no shown). The distribution of KIR2D-HLA-C1/C2 showed no difference between YNH and FYDH (data not shown). These data also showed the existence of a preponderant inhibitory KIR/HLA signaling pathway in these populations.

The extensive diversity of the HLA and KIR genes and the central role of their interaction in immune responses to different pathogens are expected to favor the coevolution of these loci as suggested in several disease, population, and comparative genetic studies across primate species [11, 31, 32]. However, in the present study, the main difference was observed for HLA but not for KIR and HLA/KIR combinations. We assumed this difference might be affected for two reasons. Firstly, the HLA genes were genotyped for SNPs at a high resolution level, allowing allelic differences to be more easily detected. Secondly, the KIR genes were detected only at the level of their presence or absence in each individual and the KIR SNPs were not genotyped. The difference between the KIR gene alleles of the YNH and FYDH might exist more obviously at the SNP level. In addition, the KIR gene cluster is located in a rapidly evolving genomic region where the activating KIR genes evolved from inhibitory KIR genes and were short lived in comparison to genes encoding the inhibitory KIR genes [33]. Our previous study as well as other population studies have suggested that the geographic distribution of populations was more closely related to activating KIR genes than to inhibitory KIR genes [16, 34]. The only difference in the KIR gene cluster observed in the present study was for the KIR2DS5. According to our epidemic data, the FYDH has been threatened with infectious diseases such as hepatitis A virus (HAV) infection with the antibody to HAV detected in more than 90% of the population. Thus, we deduced that after FYDH isolation, the activating KIR genes have evolved more rapidly than the inhibitory KIR genes in response to epidemics.

In conclusion, the current study determined the distribution of KIR/HLA gene combinations in the YNH. In combination with earlier published data, we demonstrated that the YNH population has a particular distribution of HLA alleles, which is different compared to FYDH population. However, the KIR genotypes and the combinations of KIR/HLA were similar in both Han populations. Our results will help us to understand the role of KIR/HLA in autoimmune diseases and viral infections. Epidemiology of the YNH population and the distributions of other immune system genes need to be investigated further.

## Figures and Tables

**Table 1:** The Hardy-Weinberg's equilibrium observe heterozygosity, expect heterozygosity and Ewens-Watterson test of neutrality for HLA-A, -B, and -C loci in Yunnan Han.

	Alleles (*n*)	Observe heterozygosity	Expect heterozygosity	H-W *P* value	E-W *P* value
A	27	0.858	0.861	0.148	0.609
C	31	0.920	0.941	0.162	0.355
B	54	0.866	0.900	0.255	0.528

**Table 2:** HLA-A, HLA-B, and HLA-C allele frequencies in Yunnan Han.

HLA-A	Frequency	HLA-B	Frequency	HLA-B	Frequency	HLA-C	Frequency
A*01:01	0.027	B*07:02	0.013	B*40:01	0.117	C*01:02	0.203
A∗02:01	0.086	B*07:05	0.004	B*40:02	0.010	C*01:03	0.002
A*02:03	0.040	B*08:01	0.006	B*40:06	0.019	C*01:10	0.002
A*02:04	0.006	B*13:01	0.084	B*44:02	0.013	C*02:02	0.004
A*02:05	0.002	B*13:02	0.029	B*44:03	0.027	C*03:02	0.059
A*02:06	0.052	B*14:02	0.002	B*46:01	0.144	C*03:03	0.059
A*02:07	0.094	B*15:01	0.042	B*47:01	0.002	C*03:04	0.117
A*02:10	0.002	B*15:02	0.044	B*48:01	0.021	C*03:07	0.002
A*03:01	0.025	B*15:07	0.004	B*48:03	0.004	C*04:01	0.061
A*03:02	0.002	B*15:10	0.002	B*49:01	0.002	C*04:03	0.031
A*11:01	0.268	B*15:11	0.017	B*50:01	0.004	C*04:04	0.002
A*11:02	0.006	B*15:12	0.006	B*51:01	0.031	C*05:01	0.004
A*11:05	0.002	B*15:17	0.002	B*51:02	0.006	C*05:04	0.006
A*23:01	0.004	B*15:18	0.002	B*52:01	0.031	C*05:11	0.002
A*24:02	0.190	B*15:25	0.019	B*52:05	0.002	C*06:02	0.050
A*24:03	0.004	B*15:27	0.006	B*52:06	0.002	C*06:04	0.002
A*24:07	0.006	B*27:03	0.002	B*54:01	0.040	C*07:01	0.029
A*26:01	0.019	B*27:04	0.013	B*55:02	0.025	C*07:02	0.153
A*29:01	0.004	B*27:05	0.006	B*56:01	0.002	C*07:04	0.002
A*30:01	0.031	B*27:06	0.002	B*56:02	0.004	C*08:01	0.040
A*31:01	0.017	B*27:07	0.002	B*57:01	0.006	C*08:02	0.002
A*32:01	0.010	B*35:01	0.038	B*57:08	0.002	C*08:04	0.038
A*33:01	0.002	B*35:02	0.002	B*58:01	0.054	C*08:15	0.002
A*33:03	0.086	B*35:05	0.008	B*67:01	0.004	C*12:02	0.038
A*68:01	0.006	B*35:10	0.004	B*78:01	0.004	C*12:03	0.021
A*69:01	0.004	B*37:01	0.008	B*78:02	0.002	C*12:14	0.002
A*74:01	0.002	B*38:02	0.031			C*14:02	0.038
		B*39:01	0.019			C*14:03	0.002
						C*14:06	0.002
						C*15:02	0.023
						C*15:04	0.002

**Table 3:** Global linkage disequilibrium (D′ and Wn) values in Yunnan Han.

Locus pair	D	D′	Wn	*P* value
A : B	0.005	0.608	0.581	<0.001
A : C	0.007	0.503	0.413	<0.001
B : C	0.009	0.859	0.688	<0.001

**Table 4:** Common HLA A-C-B, A-B, A-C, and B-C haplotypes in Yunnan Han.

A-C-B	Frequency	A-B	Frequency	A-C	Frequency	C-B	Frequency
A*02:01-C*01:02-B*46:01	0.011	A*02:01-B*40:01	0.013	A*01:01-C*06:02	0.014	B*07:02-C*07:02	0.013
A*02:03-C*07:02-B*38:02	0.010	A*02:01-B*46:01	0.012	A*02:01-C*01:02	0.025	B*13:01-C*03:04	0.071
A*02:07-C*01:02-B*46:01	0.067	A*02:03-B*38:02	0.010	A*02:01-C*03:04	0.015	B*13:02-C*06:02	0.023
A*11:01-C*01:02-B*46:01	0.029	A*02:07-B*40:01	0.011	A*02:01-C*07:02	0.015	B*15:01-C*04:01	0.013
A*11:01-C*01:02-B*54:01	0.012	A*02:07-B*46:01	0.073	A*02:03-C*01:02	0.010	B*15:01-C*12:03	0.010
A*11:01-C*03:04-B*13:01	0.031	A*03:01-B*44:02	0.010	A*02:03-C*07:02	0.018	B*15:02-C*08:01	0.019
A*11:01-C*03:04-B*40:01	0.018	A*11:01-B*13:01	0.044	A*02:06-C*08:01	0.010	B*15:02-C*08:04	0.017
A*11:01-C*07:02-B*40:01	0.023	A*11:01-B*15:01	0.024	A*02:07-C*01:02	0.068	B*15:11-C*03:03	0.015
A*11:01-C*08:01-B*15:02	0.014	A*11:01-B*15:02	0.027	A*11:01-C*01:02	0.050	B*15:25-C*04:03	0.019
A*11:01-C*12:02-B*52:01	0.010	A*11:01-B*38:02	0.011	A*11:01-C*03:03	0.013	B*35:01-C*03:03	0.015
A*11:01-C*14:02-B*51:01	0.013	A*11:01-B*40:01	0.042	A*11:01-C*03:04	0.049	B*35:01-C*04:01	0.019
A*24:02-C*01:02-B*46:01	0.017	A*11:01-B*46:01	0.033	A*11:01-C*07:02	0.070	B*38:02-C*07:02	0.029
A*24:02-C*01:02-B*54:01	0.013	A*11:01-B*51:01	0.015	A*11:01-C*08:01	0.019	B*39:01-C*07:02	0.019
A*24:02-C*03:04-B*13:01	0.015	A*11:01-B*54:01	0.010	A*11:01-C*08:04	0.011	B*40:01-C*03:04	0.040
A*24:02-C*04:03-B*15:25	0.017	A*11:01-B*55:02	0.012	A*11:01-C*12:02	0.014	B*40:01-C*07:02	0.052
A*24:02-C*07:02-B*40:01	0.011	A*24:02-B*13:01	0.019	A*11:01-C*12:03	0.011	B*44:03-C*07:01	0.017
A*30:01-C*06:02-B*13:02	0.021	A*24:02-B*15:25	0.017	A*11:01-C*14:02	0.010	B*46:01-C*01:02	0.132
A*33:03-C*03:02-B*58:01	0.042	A*24:02-B*35:01	0.016	A*24:02-C*01:02	0.032	B*48:01-C*08:01	0.010
A*33:03-C*07:01-B*44:03	0.013	A*24:02-B*40:01	0.035	A*24:02-C*03:03	0.016	B*51:01-C*14:02	0.025
		A*24:02-B*46:01	0.014	A*24:02-C*03:04	0.020	B*52:01-C*12:02	0.022
		A*24:02-B*54:01	0.016	A*24:02-C*04:01	0.028	B*54:01-C*01:02	0.036
		A*30:01-B*13:02	0.027	A*24:02-C*04:03	0.024	B*55:02-C*01:02	0.013
		A*33:03-B*44:03	0.016	A*24:02-C*07:02	0.034	B*58:01-C*03:02	0.054
		A*33:03-B*58:01	0.042	A*30:01-C*06:02	0.021		
				A*33:03-C*03:02	0.041		
				A*33:03-C*07:01	0.017		

**Table 5:** Two KIR3D and their ligands HLA-A3 and HLA-A11 in Yunnan Han and FYDH.

KIR3DL2-A3/A11 pair	Yunnan Han (*n* = 239)		FYDH (*n* = 93)		Yunnan Han versus FYDH
	*N*	Frequency	*N*	Frequency	*P* value
3DL2^+^-A3/A11^+^	126	0.527	53	0.570	0.540
3DL2^+^-A3/A11^−^	113	0.473	40	0.430	0.540

**Table 6:** Two KIR3D and their ligands HLA-Bw4 in Yunnan Han and FYDH.

KIR3DL1-Bw4 pair	Yunnan Han (*n* = 239)		FYDH (*n* = 92)		Yunnan Han versus FYDH
	*N*	Frequency	*N*	Frequency	*P* value
3DL1^+^-Bw4^+^	132	0.552	51	0.554	1.000
3DL1^+^-Bw4^−^	95	0.397	39	0.424	0.708
3DL1^−^-Bw4^+^	9	0.038	0	0.000	0.067
3DL1^−^-Bw4^−^	3	0.013	2	0.022	0.620

**Table 7:** Combination of two inhibitory (KIR2DL2/3 + HLA-C1 and KIR2DL1 + HLA-C2) and two activating (2DS2 + HLA-C1 and 2DS1 + HLA-C2) receptor-ligand pairs in YNH and FYDH.

Number of pairs	KIR/HLA pairs	Yunnan Han (*n* = 239)		FYDH (*n* = 93)		YNH versus FYDH
		*N*	Frequency	*N*	Frequency	*P* value
Inhibitory or activating						
None		3	0.013	0	0.000	0.562
One pair	2DL1 + HLA-C2	5	0.021	1	0.011	1.000
	2DL2/3 + HLA-C1	152	0.636	66	0.710	0.247
	2DS1 + HLA-C2	0	0.000	0	0.000	—
Two pairs	2DL1 + HLA-C2, 2DL2/3 + HLA-C1	42	0.176	19	0.204	0.532
	2DL1 + HLA-C2, 2DS1 + HLA-C2	4	0.017	0	0.000	0.580
	2DL2/3 + HLA-C1, 2DS1 + HLA-C2	0	0.000	1	0.011	0.280
Three pairs	2DL1 + HLA-C2, 2DL2/3 + HLA-C1, 2DS1 + HLA-C2	33	0.138	6	0.065	0.086
Inhibitory						
One pair	2DL2/3 + HLA-C1	152	0.636	67	0.720	0.158
	2DL1 + HLA-C2	9	0.038	1	0.011	0.294
Two pairs	2DL1 + HLA-C2, 2DL2/3 + HLA-C1	75	0.314	25	0.269	0.506
None		3	0.013	0	0.000	0.562
Activating						
One pair	2DS1 + HLA-C2	37	0.155	7	0.075	0.070
None		202	0.845	86	0.925	

## References

[1] Shiina T, Hosomichi K, Inoko H, Kulski JK (2009). The HLA genomic loci map: expression, interaction, diversity and disease. *Journal of Human Genetics*.

[2] Vilches C, Parham P (2002). KIR: diverse, rapidly evolving receptors of innate and adaptive immunity. *Annual Review of Immunology*.

[3] Middleton D, Gonzelez F (2010). The extensive polymorphism of KIR genes. *Immunology*.

[4] Lanier LL (2005). NK cell recognition. *Annual Review of Immunology*.

[5] Dohring C, Scheidegger D, Samaridis J, Cella M, Colonna M (1996). A human killer inhibitory receptor specific for HLA-A. *The Journal of Immunology*.

[6] Bashirova AA, Martin MP, McVicar DW, Carrington M (2006). The killer immunoglobulin-like receptor gene cluster: tuning the genome for defense. *Annual Review of Genomics and Human Genetics*.

[7] Pende D, Biassoni R, Cantoni C (1996). The natural killer cell receptor specific for HLA-A allotypes: a novel member of the p58/p70 family of inhibitory receptors that is characterized by three immunoglobulin-like domains and is expressed as a 140-kD disulphide- linked dimer. *The Journal of Experimental Medicine*.

[8] Gumperz JE, Litwin V, Phillips JH, Lanier LL, Parham P (1995). The Bw4 public epitope of HLA-B molecules confers reactivity with natural killer cell clones that express NKB1, a putative HLA receptor. *The Journal of Experimental Medicine*.

[9] Cella M, Longo A, Ferrara GB, Strominger JL, Colonna M (1994). NK3-specific natural killer cells are selectively inhibited by Bw4- positive HLA alleles with isoleucine 80. *The Journal of Experimental Medicine*.

[10] Carr WH, Pando MJ, Parham P (2005). KIR3DL1 polymorphisms that affect NK cell inhibition by HLA-Bw4 ligand. *The Journal of Immunology*.

[11] Parham P (2005). MHC class I molecules and KIRS in human history, health and survival. *Nature Reviews Immunology*.

[12] Kim S, Sunwoo JB, Yang L (2008). HLA alleles determine differences in human natural killer cell responsiveness and potency. *Proceedings of the National Academy of Sciences of the United States of America*.

[13] Shi L, Xu SB, Ohashi J (2006). HLA-A, HLA-B, and HLA-DRB1 alleles and haplotypes in Naxi and Han populations in Southwestern China (Yunnan province). *Tissue Antigens*.

[14] Yao Y, Shi L, Shi L (2009). Distribution of HLA-A, -B, -Cw, and -DRB1 alleles and haplotypes in an isolated Han population in Southwest China. *Tissue Antigens*.

[15] Shi L, Shi L, Tao Y (2011). Distribution of killer cell immunoglobulin-like receptor genes and combinations with HLA-C ligands in an isolated Han population in Southwest China. *Tissue Antigens*.

[16] Shi L, Zhang H, Shen Y (2013). Distribution of KIR genes in Han population in Yunnan Province: comparison with other Han populations in China. *International Journal of Immunogenetics*.

[17] Lancaster A, Nelson MP, Meyer D, Thomson G, Single RM (2003). PyPop: a software framework for population genomics: analyzing large-scale multi-locus genotype data. *Pacific Symposium on Biocomputing*.

[18] Lancaster AK, Single RM, Solberg OD, Nelson MP, Thomson G (2007). PyPop update—a software pipeline for large-scale multilocus population genomics. *Tissue Antigens*.

[19] Gutierrez JP, Royo LJ, Alvarez I, Goyache F (2005). MolKin v2.0: a computer program for genetic analysis of populations using molecular coancestry information. *The Journal of Heredity*.

[20] Guo SW, Thompson EA (1992). Performing the exact test of Hardy-Weinberg proportion for multiple alleles. *Biometrics*.

[21] Ewens WJ (1972). The sampling theory of selectively neutral alleles. *Theoretical Population Biology*.

[22] Watterson GA (1978). The homozygosity test of neutrality. *Genetics*.

[23] You Z (1994). *History of Yunnan Nationalities*.

[24] Layrisse Z, Guedez Y, Dominguez E (2001). Extended HLA haplotypes in a Carib Amerindian population: the Yucpa of the Perija Range. *Human Immunology*.

[25] Chen S, Hu Q, Liu Z (2007). The distribution of HLA alleles revealed a founder effect in the geographically isolated Chinese population, Drung. *Molecular immunology*.

[26] Flores AC, Marcos CY, Paladino N (2007). KIR genes polymorphism in Argentinean Caucasoid and Amerindian populations. *Tissue Antigens*.

[27] Velickovic M, Velickovic Z, Dunckley H (2006). Diversity of killer cell immunoglobulin-like receptor genes in Pacific Islands populations. *Immunogenetics*.

[28] Yawata M, Yawata N, McQueen KL (2002). Predominance of group A KIR haplotypes in Japanese associated with diverse NK cell repertoires of KIR expression. *Immunogenetics*.

[29] Whang DH, Park H, Yoon JA, Park MH (2005). Haplotype analysis of killer cell immunoglobulin-like receptor genes in 77 Korean families. *Human Immunology*.

[30] Rajalingam R, Du Z, Meenagh A (2008). Distinct diversity of KIR genes in three Southern Indian populations: comparison with world populations revealed a link between KIR gene content and pre-historic human migrations. *Immunogenetics*.

[31] Single RM, Martin MP, Gao X (2007). Global diversity and evidence for coevolution of KIR and HLA. *Nature Genetics*.

[32] Carrington M, Martin MP (2006). The impact of variation at the KIR gene cluster on human disease. *Current Topics in Microbiology and Immunology*.

[33] Abi-Rached L, Parham P (2005). Natural selection drives recurrent formation of activating killer cell immunoglobulin-like receptor and Ly49 from inhibitory homologues. *The Journal of Experimental Medicine*.

[34] Middleton D, Meenagh A, Moscoso J, Arnaiz-Villena A (2008). Killer immunoglobulin receptor gene and allele frequencies in Caucasoid, Oriental and Black populations from different continents. *Tissue Antigens*.

